# Circulating tumour cells in the central and the peripheral venous compartment in patients with metastatic breast cancer

**DOI:** 10.1038/bjc.2011.122

**Published:** 2011-04-05

**Authors:** D J E Peeters, G G Van den Eynden, P-J van Dam, A Prové, I H Benoy, P A van Dam, P B Vermeulen, P Pauwels, M Peeters, S J Van Laere, L Y Dirix

**Affiliations:** 1 Translational Cancer Research Group, Laboratory of Pathology, Antwerp University/Oncology Centre, GZA Hospitals St-Augustinus, Antwerp 2610, Belgium; 2 Laboratory for Molecular Biology, Labo Lokeren, Campus RIATOL, Antwerp 2000, Belgium; 3 Department of Pathology, Antwerp University/Antwerp University Hospital, Antwerp 2610, Belgium; 4 Department of Oncology, Antwerp University/Antwerp University Hospital, Antwerp 2610, Belgium

**Keywords:** circulating tumour cells, metastatic breast cancer, CellSearch system, peripheral venous blood, central venous blood

## Abstract

**Background:**

The enumeration of circulating tumour cells (CTC) has prognostic significance in patients with metastatic breast cancer (MBC) and monitoring of CTC levels over time has considerable potential to guide treatment decisions. However, little is known on CTC kinetics in the human bloodstream.

**Methods:**

In this study, we compared the number of CTC in both 7.5 ml central venous blood (CVB) and 7.5 ml peripheral venous blood (PVB) from 30 patients with MBC starting with a new line of chemotherapy.

**Results:**

The number of CTC was found to be significantly higher in CVB (median: 43.5; range: 0–4036) than in PVB (median: 33; range: 0–4013) (*P*=0.001). When analysing samples pairwise, CTC counts were found to be significantly higher in CVB than in PVB in 12 out of 26 patients with detectable CTC. In contrast, only 2 out of 26 patients had higher CTC counts in PVB as compared with CVB, whereas in 12 remaining patients no significant difference was seen. The pattern of CTC distribution was independent of the sites of metastatic involvement.

**Conclusion:**

A substantial difference in the number of CTC was observed between CVB and PVB of patients with MBC. Registration of the site of blood collection is warranted in studies evaluating the role of CTC assessment in these patients.

Over the past 10 years, a vast body of evidence has emerged supporting the use of circulating tumour cells (CTC) as a promising, new prognostic factor in the clinical management of patients with metastatic breast, colorectal and prostate cancer (Cristofanilli *et al*, 2004; Cohen *et al*, 2008; De Bono *et al*, 2008). Several studies strongly suggest an equal role in early breast cancer (Ignatiadis *et al*, 2008; Pierga *et al*, 2008; Rack *et al*, 2010). In addition, molecular characterisation of CTC has been shown to have the potential to serve as a powerful pharmacodynamic marker to predict tumour sensitivity to specific targeted treatments (Meng *et al*, 2004b; de Bono *et al*, 2007; Attard *et al*, 2009).

Several approaches for the detection, enumeration and isolation of CTCs in blood samples of patients with various types of cancer have been developed (Mostert *et al*, 2009). Only the CellSearch System (Veridex, Raritan, NJ, USA), a semi-automated system for the immunomagnetic enrichment of CTC based on their expression of epithelial cell adhesion molecule (EpCAM), immunolabeling and image cytometry, has gained approval of the US Food and Drug Administration (FDA). With this technique two or more CTC per 7.5 ml blood are detected in ca. 60% of patients with metastatic breast cancer (MBC). Furthermore, a cutoff of five CTC or more per 7.5 ml of blood has been applied to distinguish between good and worse prognosis in patients with MBC (Cristofanilli *et al*, 2004; Hayes *et al*, 2006).

Previous studies have addressed several analytical sources of variability in CTC measurements by the CellSearch System, however, little is known about the biological variability and kinetics of CTC in the human bloodstream (Allard *et al*, 2004; Riethdorf *et al*, 2007; Tibbe *et al*, 2007; Kraan *et al*, 2011). Given the physical mismatch between malignant epithelial cells, typically having a diameter of 10–30 *μ*m, and human capillaries, on average only 3–8 *μ*m in diameter ((Boulpaep, 2003) (p. 464)), considerable variability in the number of CTC throughout the circulation, both in time and space, can be anticipated. Experiments in mouse models have shown that CTC transiently peak in the circulation after injection in the tail vein or left cardiac ventricle, after which they steadily increase in parallel with the development of micrometastases in lymph nodes and/or bone marrow over several weeks of time (Goodale *et al*, 2009). In line with these observations, CTC have been shown to disappear from the bloodstream in the majority of patients with localised breast cancer within days after surgical removal of the primary tumour (Krag *et al*, 1999; Meng *et al*, 2004a; Biggers *et al*, 2009; Sandri *et al*, 2010). Furthermore, for most solid malignancies typical patterns of metastasis can be predicted based on the anatomical localisation of the primary tumour (Weiss, 1992). In a recent study evaluating the impact of different surgical interventions for colorectal liver metastases on CTC levels in anatomically discrete vascular compartments, CTC appeared to be localised more abundantly to the hepatic macrocirculation whereas significantly fewer enter the peripheral circulation, suggesting that the liver and the lungs act as the major site of retention for CTC in these patients (Jiao *et al*, 2009).

With this study, we wanted to investigate anticipated differences in the occurrence, number and characteristics of CTC in different vascular compartments of patients with breast cancer. As quantitative cutoff values in the number of CTC are used to distinguish between patients with good and worse prognosis, differences in the number of CTC according to the site of blood sampling might be of direct clinical importance. Furthermore, differences in numbers and in characteristics of CTC in different vascular compartments might help to gain insight into the biology of these cells and the metastatic process.

## Materials and Methods

### Patients and sample collection

Consecutive patients with MBC with either untreated metastatic disease or progressive metastatic disease before the start of a new line of treatment, who had an implanted central venous vascular access system, were recruited from November 2009 till November 2010. Appropriate local ethics committee approval was obtained and written informed consent was obtained from all patients.

### CTC enumeration and characterisation

The CTC were isolated and enumerated using the CellSearch System (Veridex) in both 7.5 ml central venous blood (CVB) – obtained from the implanted vascular device – and 7.5 ml peripheral venous blood (PVB) – obtained from an antecubital vein. Both blood samples were drawn simultaneously in CellSave Preservative Tubes (Immunicon Inc., Huntingdon Valley, PA, USA), stored at room temperature and processed within 72 h. Paired samples were analysed in parallel using the CellSearch Circulating Tumour Cell kit (Veridex) according to the manufacturer's instructions. Criteria for an EpCAM positive object to be identified as a CTC include a round-to-oval morphology, a visible nucleus (DAPI+), positive staining for cytokeratin and negative staining for CD45. Each sample was analysed independently by two readers (DP and GVdE). Questionable interpretations were evaluated again until consensus was reached. As a measure for the size of CTC, the geometrical area of each individual cell was estimated based on the two longest perpendicular diameters according to the following formula: *π* × A/2 × B/2 (with A and B being the two longest perpendicular diameters measured on screen) (Figure 2A).

### Statistical analysis

Statistical analyses were performed using SPSS 16.0 software (SPSS Inc., Chicago, IL, USA). A two-sided *P*⩽0.05 was considered to be statistically significant. Normality was tested with a Kolmogorov–Smirnov test, assuming normality of data if *P*>0.1. In the case of normal distribution in all subgroups, equality of means was tested using Student's *t*-test. The Mann–Whitney *U*-test, Kruskal–Wallis test or Wilcoxon Signed Ranks test were used to assess differences between non-parametric distributed variables. Correlations between continuous variables were analysed with Spearman's correlation statistics. The Pearson's *χ*^2^-test was used to assess the relation between categorical variables.

## Results

A total of 30 patients were recruited into this study. This group comprised of four patients with primary MBC and 26 patients with MBC progressing under treatment. The median age of the patient population was 62 (range: 40–85) years. Almost all patients had diffuse metastatic involvement and most patients with progressive metastatic disease were extensively pretreated with multiple lines and types of systemic treatments. Other clinicopathological data are summarised in Table 1.

### Comparison of the number of CTC in different vascular compartments

**Figure 1:**
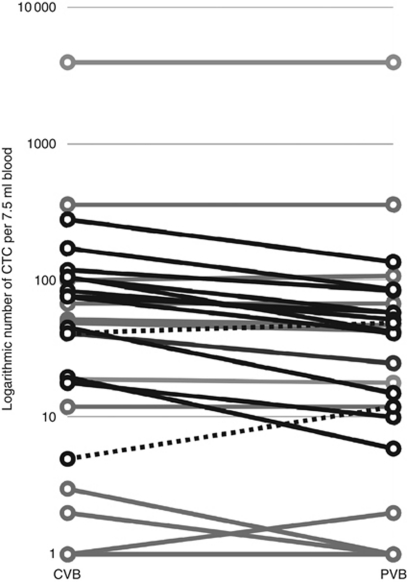
Logarithmic number of CTC per 7.5 ml of CVB and PVB of the 30 MBC patients. Sample code: bold=#CTC_CVB_>#CTC_PVB_ (Table 2, group 1); dashed=#CTC_CVB_<#CTC_PVB_ (Table 2, group 3); grey=#CTC_CVB_=#CTC_PVB_ (Table 2, group 2).

The number of CTC was measured in different vascular compartments. In all patients CTC were assessed in CVB and PVB. Figure 1 and Table 2 represent the results. In four (13%) patients no CTC were found in any of the vascular compartments. The median number of CTC was 43.5 (range: 0–4036) in CVB and 33 (range: 0–4013) in PVB (*P*=0.001). The CTC counts in CVB and PVB were strongly correlated (*R*^2^=0.952, *P*<0.001). Both CTC counts in CVB and PVB were correlated with levels of CA15.3 (*R*^2^_CVB_=0.498, *P*=0.005; *R*^2^_PVB_=0.509, *P*=0.004). Except for a higher number of CTC in CVB in the presence of bone metastasis (*P*=0.044), no correlations between the number of CTC in either CVB or PVB and specific sites of metastatic involvement were observed.

Using the five CTC per 7.5 ml prognostic cutoff previously described (Cristofanilli *et al*, 2004) to discriminate between CTC positive (⩾5 CTC per 7.5 ml) and CTC negative (<5 CTC per 7.5 ml) MBC patients, 22 out of 30 (73%) patients were CTC positive for both CVB and PVB. There was a 100% concordance between the results of the CVB and PVB (Table 2).

### Comparison of CTC distribution patterns

We subsequently divided the 26 patients with detectable CTC in three distinct groups based on the difference in CTC counts between paired CVB and PVB samples, calculated as the mathematical difference of both counts divided by their mean. A threshold of 15% was applied to define a significant difference based on coefficients of variation for different sources of analytical variability of the CellSearch System reported in literature (Allard *et al*, 2004; Riethdorf *et al*, 2007; Kraan *et al*, 2011). Because of statistical considerations, patients with less than five CTC per 7.5 ml in both CVB and PVB were considered to have equal counts in both compartments. Using these criteria 12 out of 26 patients (46%) patients had significantly higher CTC counts in CVB than in PVB with a median percentage of difference of 59% (range: 26–108%) whereas in only two (8%) patients higher numbers were observed in PVB with a median percentage of difference of 50% (range: 18–82%). In the remaining 12 (46%) patients no difference was observed between both counts (Table 2). No correlation was found between the pattern of CTC distribution in CVB and PVB and specific sites of metastasis in general or the prevalence of clinically evident lung metastases in particular.

### Comparison of CTC characteristics at different vascular compartments

**Figure 2:**
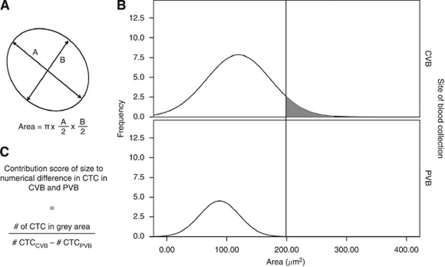
Comparison of CTC size in CVB and PVB. (**A**) Calculation method for the area of an individual CTC as a measure for size. (**B**) Frequency curves of CTC sizes in CVB and PVB for one illustrative patient. A cutoff for size in CVB was applied at the maximal size measured in PVB. (**C**) Formula for the calculation of the ‘contribution score’ for size to the numerical difference in CTC between CVB and PVB.

We compared the size of the CTC at different vascular compartments. CTC were measured on screen in two dimensions and size was estimated based on the geometrical area of an ellipse as shown in Figure 2. Mean size of CTC in CVB and PVB of the same patient was compared only in those patients with at least five measurable CTC in both compartments. Overall, mean CTC area measured 77.59±4.68 *μ*m^2^ in CVB and 62.28±5.02 *μ*m^2^ in PVB, respectively (*P*<0.001). When analysing samples pairwise, CTC measured in CVB were significantly larger than CTC measured in PVB in 11 out of 22 patients (50%). In the other 11 patients no statistically significant difference in average CTC size between CVB and PVB was observed. Furthermore, we calculated a score to estimate the contribution of size to the numerical difference in CTC counts between CVB and PVB as demonstrated in Figure 2B and C. When applying a cutoff for CTC size in CVB at the maximal CTC size measured in PVB, on average 19% (range: 0–48%) of the numerical difference in CTC counts between CVB and PVB could be explained on the basis of size (Table 2; Figure 2C).

## Discussion

In this study, we compared the number of CTC, as assessed by the CellSearch System, in blood samples obtained from different sites throughout the circulation in 30 patients with MBC. We observed significantly higher numbers of CTC in CVB than in PVB in 46% of the patients with detectable CTC. The opposite was true in only 8% of the patients whereas in another 46% of patients no difference was observed at all. Given the fact that all patients included in this study suffered from extensive metastatic involvement, the observation of a steep concentration gradient of CTC in the blood circulation from the central venous compartment towards the peripheral venous compartment is in accordance with the differences in CTC counts between portosystemic and peripheral systemic circulations in patients with liver metastasis from colorectal cancer reported by Jiao *et al* (2009).

We believe that the findings of the current study have several important clinical and biological implications. First, although it was generally reassuring that a 100% concordance between CVB and PVB was observed when CTC results where dichotomised according to the ⩾5 CTC per 7.5 ml prognostic cutoff, differing CTC counts according to the site of blood collection might be confounding when using decreases or increases in absolute CTC numbers as a surrogate endpoint for the assessment of treatment efficacy in cancer patients as proposed by several authors (Smith *et al*, 2000; Pachmann *et al*, 2008; Molife *et al*, 2011). From this perspective, comparison of CTC levels over the course of treatment would only render reliable information if blood samples were obtained from the same vascular site. This also implies the need of recording the site of blood sampling on every occasion. In addition, as this was only a small study it cannot be excluded that in larger patient series discordant results according to the ⩾5 CTC per 7.5 ml prognostic cutoff will be observed.

Second, the observed difference in CTC numbers between CVB and PVB might also provide direct insight into CTC biology in the human circulation. As most patients included in this study had multiple systemic metastases – draining directly to the CVB compartment – and metastases in the distal arm – draining to the antecubital vein from which the PVB was drawn – were not seen, the observation of higher CTC numbers in the CVB compared with the PVB suggests an important filtering function for the lung microvascular system in these patients. Although this might not apply for CTC shedding from lung metastases, we were not able to show any association between the CTC distribution pattern and the presence or absence of clinically evident lung metastases, probably because almost all patients with lung metastases also suffered from diffuse systemic disease. Only one patient was diagnosed with isolated lung metastases. In this patient one CTC was observed, both in CVB and PVB.

Taking into account that the average cardiac output of a resting adult is ∼5 l per min and assuming that the difference in CTC counts between CVB and PVB is constant over time, one could calculate the total number of CTC filtered by the lungs in 24 h. For instance for patient 1 in Table 2 presenting with 175 CTC per 7.5 ml in CVB and 87 CTC per 7.5 ml in PVB, this would mean that a total of 85 million CTC would be filtered out by the lungs in 1 day's time. The cumulative entrapment of CTC in the microcirculation of the lungs might potentially provide an explanation for some radiologically unexplained respiratory distress syndromes frequently observed in end-stage cancer patients (Roberts *et al*, 2003). The observation of intravascular tumour cells in microvascular blood samples in patients suggested to suffer from so-called ‘lymphangitic’ carcinomatosis is in keeping with this observation (Masson *et al*, 1989).

Third, to address the extent to which size filtration might contribute to the observed differences in more depth, we compared the size of CTC visualised by the CellSearch System in CVB and PVB of the same patient. Although in 50% of the patients CTC in CVB were significantly larger than CTC in PVB, these size differences could only explain 0–48% of the difference in CTC counts observed between both compartments.

Last, the lung is a very frequent site of metastatic growth in patients with MBC. Animal models have suggested that a primary intravascular location of breast cancer cells occurs as a mode of intravascular growth before extravasation (Wong *et al*, 2002). Our observation of a high degree of cancer cell retention in the lung, suggests at least a stochastic advantage for the lung to harbour efficient metastatic growth. Again, this is in keeping with the observations made by Jiao *et al* (2009) explaining the high propensity of colorectal cancer to seed to the liver and lungs. It must be stressed that these observations are by no means in contradiction with any superimposed selective and organ-specific homing model that, beside size filtration, might also account in part for the loss of (subpopulations) of CTC throughout the circulation (Müller *et al*, 2001; Glinskii *et al*, 2005; Nguyen *et al*, 2009). From this perspective, it will be of interest to compare molecular profiles of CTC harvested at different sites in the blood circulation with regard to cell adhesion pathways, epithelial-mesenchymal transition and stem cell characteristics, which is the subject of an ongoing study.

A limitation of the current study is the variety of lines and types of treatment the included patients previously had received. Rather than representing one cell population, CTC are considered to form a heterogeneous cell population and different treatments might have discrete effects on specific CTC subpopulations (Meng *et al*, 2004b; Aktas *et al*, 2009; Van Der Auwera *et al*, 2010). Also, the fact of most patients suffering from diffuse metastatic disease makes it very difficult to draw firm conclusions on how CTC circulate or rather shed in the blood stream in relation to specific sites of disease involvement. To address these questions properly, larger studies including more uniform, less extensively pretreated patient populations and patients with metastatic disease confined to either the lungs or the systemic circulation, must be carried out.

In conclusion, we observed statistically different numbers of CTC in CVB and PVB in 14 of 26 (54%) patients with MBC. As quantitative assessment of CTC is proposed for the evaluation of treatment efficacy in patients with MBC, registration of the site of blood collection is warranted in clinical practice and studies evaluating the role of CTC assessment to be able to draw correct conclusions.

## Figures and Tables

**Table 1:** Clinicopathological variables

**Characteristics**	***n*=30**
*Histology*	
Invasive ductal carcinoma	27 (90%)
Invasive lobular carcinoma	3 (10%)
	
*Histological grade* a	
I	4 (13%)
II	10 (33%)
III	16 (54%)
	
*Hormonal status*	
ER and/or PR positive	25 (83%)
Negative for both	5 (17%)
	
*HER2/neu status* b	
Negative	24 (80%)
Positive	4 (13%)
	
*Triple negative*	4 (13%)
	
*Number of organs involved*	
1	4 (13%)
2–5	26 (87%)
	
*Metastatic sites*	
Bone	26 (87%)
Lung	10 (33%)
Liver	20 (67%)
Central nervous system	5 (17%)
Other^c^	16 (53%)

Abbreviations: ER, oestrogen receptor; PR, progeterone receptor.

a Tumours were histologically graded according to the Nottingham modification of the Bloom and Richardson histologic grading scheme.

b HER2/neu status was evaluated using immunohistochemistry (IHC) and FISH. Samples with IHC score 0/1+ or IHC 2+ and FISH− were called negative, samples with IHC score 3+ or IHC score 2+ and FISH+ were called positive. In two patients, HER2/neu status could not be determined because of insufficient tissue material.

c These sites included locoregional involvement, pleura, peritoneum, skin, lymph nodes, adrenal gland and ovary.

**Table 2:** Number of CTC in CVB and PVB of patients with metastatic breast cancer

**Patient**	**#CTC per 7.5 ml CVB**	**#CTC per 7.5 ml PVB**	**Percentage of difference between CVB and PVB** a	**Groups based on percentage of difference** b	**Contribution score of size**
1	175	87	67	1	5
2	0	0	NA	2	NA
3	77	41	61	1	8
4	108	42	88	1	21
5	19	18	5	2	NA
6	18	10	57	1	25
7	121	85	35	1	28
8	46	15	102	1	16
9	3	1	100	2	NA
10	282	138	69	1	6
11	0	0	NA	2	NA
12	49	43	13	2	NA
13	84	52	47	1	31
14	41	49	−18	3	−138
15	52	47	10	2	NA
16	105	59	56	1	48
17	20	6	108	1	14
18	100	109	−9	2	NA
19	77	59	26	1	0
20	12	12	0	2	NA
21	365	363	1	2	NA
22	1	1	0	2	NA
23	5	12	−82	3	0
24	2	1	67	2	NA
25	0	0	NA	2	NA
26	41	25	48	1	38
27	1	2	−67	2	NA
28	0	0	NA	2	NA
29	69	69	0	2	NA
30	4036	4013	1	2	NA

Abbreviations: CTC, circulating tumour cell; CVB, central venous blood; NA, not applicable; PVB, peripheral venous blood.

a Percentage of difference is defined as the mathematical difference between CTC in CVB and PVB divided by their mean.

b Groups based on the percentage of difference are defined as follows: Group 1, patients with ⩾5 CTC per 7.5 ml blood and >15% difference between CVB and PVB with CTC_CVB_>CTC_PVB_; Group 2, patients with <5 CTC per 7.5 ml blood or patients with ⩾5 CTC per 7.5 ml blood and ⩽15% difference between CVB and PVB; Group 3, patients with ⩾5 CTC per 7.5 ml blood and >15% difference between CVB and PVB with CTC_CVB_<CTC_PVB_.
